# The Murine Model of Mucopolysaccharidosis IIIB Develops Cardiopathies over Time Leading to Heart Failure

**DOI:** 10.1371/journal.pone.0131662

**Published:** 2015-07-06

**Authors:** Gabriele Giacomo Schiattarella, Giuliana Cerulo, Valeria De Pasquale, Pasquale Cocchiaro, Orlando Paciello, Luigi Avallone, Maria Paola Belfiore, Francesca Iacobellis, Daniele Di Napoli, Fabio Magliulo, Cinzia Perrino, Bruno Trimarco, Giovanni Esposito, Paola Di Natale, Luigi Michele Pavone

**Affiliations:** 1 Department of Molecular Medicine and Medical Biotechnology, University of Naples Federico II, Naples, Italy; 2 Department of Advanced Biomedical Sciences, University of Naples Federico II, Naples, Italy; 3 Department of Veterinary Medicine and Animal Productions, University of Naples Federico II, Naples, Italy; 4 Radiology Department, Second University of Naples, Naples, Italy; 5 Biotechnology Centre, AORN Cardarelli, Naples, Italy; University of Pennsylvania, UNITED STATES

## Abstract

Mucopolysaccharidosis (MPS) IIIB is a lysosomal disease due to the deficiency of the enzyme α-N-acetylglucosaminidase (NAGLU) required for heparan sulfate (HS) degradation. The disease is characterized by mild somatic features and severe neurological disorders. Very little is known on the cardiac dysfunctions in MPS IIIB. In this study, we used the murine model of MPS IIIB (NAGLU knockout mice, NAGLU^-/-^) in order to investigate the cardiac involvement in the disease. Echocardiographic analysis showed a marked increase in left ventricular (LV) mass, reduced cardiac function and valvular defects in NAGLU^-/-^ mice as compared to wild-type (WT) littermates. The NAGLU^-/-^ mice exhibited a significant increase in aortic and mitral annulus dimension with a progressive elongation and thickening of anterior mitral valve leaflet. A severe mitral regurgitation with reduction in mitral inflow E-wave-to-A-wave ratio was observed in 32-week-old NAGLU^-/-^ mice. Compared to WT mice, NAGLU^-/-^ mice exhibited a significantly lower survival with increased mortality observed in particular after 25 weeks of age. Histopathological analysis revealed a significant increase of myocardial fiber vacuolization, accumulation of HS in the myocardial vacuoles, recruitment of inflammatory cells and collagen deposition within the myocardium, and an increase of LV fibrosis in NAGLU^-/-^ mice compared to WT mice. Biochemical analysis of heart samples from affected mice showed increased expression levels of cardiac failure hallmarks such as calcium/calmodulin-dependent protein kinase II, connexin43, α-smooth muscle actin, α-actinin, atrial and brain natriuretic peptides, and myosin heavy polypeptide 7. Furthermore, heart samples from NAGLU^-/-^ mice showed enhanced expression of the lysosome-associated membrane protein-2 (LAMP2), and the autophagic markers Beclin1 and LC3 isoform II (LC3-II). Overall, our findings demonstrate that NAGLU^-/-^ mice develop heart disease, valvular abnormalities and cardiac failure associated with an impaired lysosomal autophagic flux.

## Introduction

Mucopolysaccharidoses (MPS) are a group of lysosomal storage diseases characterized by the accumulation of glycosaminoglycans (GAGs) in various organs of affected patients [1]. The storage defect is due to the absence of lysosomal enzymes involved in GAG catabolism. Deposition of GAGs in the heart of individuals with MPS causes cardiac dysfunctions [2]. Although advances in MPS treatment, including enzyme replacement therapy [3], hematopoietic stem cell transplantation [4] and gene therapy [5], have significantly improved the outcome of these disorders, death from cardiac causes continues to be common among these patients. The cardiac valves, usually mitral and aortic, the heart muscle itself and coronary arteries are characteristically affected in MPS patients [6–9]. However, the onset and extent of cardiac involvement varies depending upon the type of MPS. Children with MPS I show the earliest and most severe cardiac disorders, whereas cardiac involvement in individuals with MPS VII has been reported in adulthood [6] as well as in young individuals [10]. While cardiac involvement in patients with MPS I, II and VI has been well described [9], less is known about heart dysfunctions in MPS III.

MPS III includes four distinct diseases (A, B, C, D) due to the deficiency of enzymes involved in heparan sulfate (HS) degradation; in particular, MPS IIIB (Sanfilippo type B syndrome) is due to the deficiency of the lysosomal enzyme α-N-acetylglucosaminidase (NAGLU). Patients with MPS IIIB are characterized by profound mental retardation, behavioral problems and death usually in the second decade, along with somatic manifestations that are highly variable among the different phenotypes. Few studies have been reported on cardiac disorders in MPS IIIB patients [2, 8].

In order to get more insight into cardiac involvement in MPS IIIB disease, in this study we used the murine model of the disease obtained by NAGLU gene disruption (NAGLU knockout mice, NAGLU^-/-^) [11]. These mice exhibit a massive increase in HS deposition in the liver and kidney, and, at a lesser extent, in the lung, spleen, thymus and heart. Here, we investigated cardiac morphology and function in NAGLU^-/-^ mice compared to wild-type (WT) littermates over time using cardiac ultrasound, and histological and biochemical analyses. Furthermore, as an impairment of autophagy was found in embryonic fibroblasts and brain tissues from MPS IIIA mice [12], in human skin fibroblasts from MPS VI patients and in the liver, spleen, and kidney tissues from MPS VI rats [13], we also investigated the autophagic marker levels in the heart tissues of NAGLU^-/-^ mice in order to verify whether abnormal autophagy might be involved in heart dysfunction in MPS IIIB.

## Materials and Methods

### Animals and Ethics Statement

Wild-type C57/BL6 mice (WT, n = 30) were purchased from the Jackson Laboratory. NAGLU knockout mice (NAGLU^-/-^, n = 30) were obtained and genotyped as previously described [11, 14]. The mice included in the study were housed with no more than 5 per cage, maintained under identical conditions of temperature (21±1°C), humidity (60±5%) and light/dark cycle, and had free access to normal mouse chow. All experiments involving animals were conducted with a protocol specifically approved for this study by the Animal Care and Use Committee of the "Biotechnology Centre", AORN Cardarelli (Naples, Italy) and in accordance with the principles and procedures outlined in the Guide for the Care and Use of Laboratory Animals published by the US National Institutes of Health (NIH Publication No. 85-23, revised 1996).

For ultrasound echocardiografic studies, the mice (WT, n = 15 and NAGLU^-/-^, n = 15) were anesthetized with an intraperitoneal (i.p.) injection of Tiletamine 5 ml/kg, Zolazepam 5 ml/kg (Zoletil 100) and Xylazine 5 mg/Kg (Sigma-Aldrich) in order to minimize any suffering of the animals. For histological and biochemical analyses, immediately after echocardiographic imaging, mice were anesthetized with an additional i.p. injection of the above drugs, and then euthanized by cervical dislocation in compliance with the recommendations contained in the American Veterinary Medical Association (AVMA) Guidelines for the Euthanasia of Animals. Hearts were collected, and processed as reported in the following paragraphs.

Regarding the survival study, 15 WT and 15 NAGLU^-/-^ mice were used. We have determined that the humane endpoint of MPS IIIB mice varied from 8 to 12 months of age. We monitored the conditions of the mice every day, at 12:00, and if during the monitoring period the mice showed the symptoms of late-stage clinical manifestation, such as urine retention, rectal prolapse, protruding penis and spinal curvature, they were anesthetized with an i.p. injection of the above drugs, and then humanely sacrificed by cervical dislocation in compliance with the recommendations contained in the American Veterinary Medical Association (AVMA) Guidelines for the Euthanasia of Animals, to minimize suffering. Wild-type control mice were observed until they were 8 month old.

### Transthoracic echocardiography

Cardiac function of MPS IIIB animals was non-invasively monitored by transthoracic echocardiography using the Vevo 2100 high-resolution imaging system (VisualSonics). Briefly, the mice were anesthetized, and echocardiograms were performed with a 30-MHz RMV-707B scanning head. Annulus dimensions were obtained in the parasternal long-axis view during systole for semilunar valves, and the apical four-chamber view during diastole for atrioventricular valves as previously described [15]. The aortic root measurements were obtained in a modified parasternal long-axis view during diastole. Doppler interrogation was performed on the atrioventricular valve inflow in the apical four-chamber view, and semilunar valve outflow in the parasternal long axis view to assess for stenosis and regurgitation using a sample volume toggle to optimize the angle of interrogation. A modified parasternal long-axis view was required in some cases to ensure ascertainment of the maximum velocity. Cardiac chamber dimensions were measured, and ventricular function assessed from two-dimensional-directed M-mode echocardiographic images obtained from the parasternal short-axis view, and Doppler images were obtained from the apical four chamber view in accordance with consensus guidelines [16]. All measurements were obtained in triplicate and averaged.

### Histology

Whole hearts isolated from 32-week-old mice (NAGLU^-/-^, n = 5 and WT, n = 5) were fixed overnight in a bath of 4% aqueous buffered formalin, and processed for paraffin embedding. Coronal sections (10 μm thick) containing both right and left ventricles were processed as previously described [17–18]. Sequential sections from each heart were stained with haematoxylin and eosin (H&E) (Sigma-Aldrich), Masson's trichrome dye (Sigma-Aldrich) and periodic acid-Schiff Alcian blue-PAS (Dako). All sections were examined on a light microscope (Leitz, DIAPLAN), and images were acquired with a digital camera (Digital JVC, TK-C1380).

Plastic embedded sections were obtained from heart tissue specimens of 32-week-old mice fixed in 2.5% glutaraldehyde and postfixed in 1% osmium tetroxide in cacodylate buffer (0.2 M, pH 7.4). Tissues were washed and dehydrated in a graded series of ethanol solutions, cleared in propylene oxide, and embedded in Epon—Araldite resin. Semithin (0.5 μm) sections were stained with toluidine blue and examined under a light microscope.

### Immunohistochemistry

Immunostaining was performed on heart sections of NAGLU^-/-^ and WT mice using the following dilution of the primary mouse monoclonal antibodies: CD3, 1:50 (Dako, clone F7.2.38); CD4, 1:15 (Vision Bio-System, clone IF6); CD8, 1:30 (Dako, clone C8/144B) and CD68, 1:50 (Dako, clone KP-1). Paraffin-embedded tissue specimens were cut at 4 μm thick sections, mounted on microscope slides, deparaffinized and then re-dehydrated. The deparaffinized slides were then boiled by microwave for 12 min in citrate buffer (pH 6) and stained with primary antibodies over night. The immunoreactions were visualized using the mouse version of the EnVision+ system (Dako) and diaminobenzidine (DAB). Sections were counterstained with Mayer’s haematoxylin. In the corresponding negative control sections, the primary antibody was either omitted or replaced with normal serum.

### RNA extraction and real-time PCR

Total RNA was extracted from several pieces of left ventricle (LV) frozen samples obtained from the whole hearts of 32-week-old mice (NAGLU^-/-^, n = 5 and WT, n = 5) using the TRIzol (Invitrogen) according to the manufacturer’s instruction. Oligo-dT first strand cDNAs were synthesized using the SuperScript VILO cDNA Synthesis (Life Technologies) according to the manufacturer’s instructions. mRNA expression was determined by quantitative real-time PCR (RT-PCR) as previously described [19–20], using an IQ-5 multicolor RT-PCR detection system (Bio-Rad) and a SYBR Green PCR Master Mix (Bio-Rad). The primers used were: myosin heavy polypeptide 7 (Myh7, β-MHC): forward 5’-CGGAAACTGAAAACGGAAAG-3’, reverse 5’-TCCTCGATCTTGTCGAACTTG-3’; atrial natriuretic peptide (ANP): forward 5’-CACAGATCTGATGGATTTCAAGA-3’, reverse 5’-CCTCATCTTCTACCGGCATC-3’; brain natriuretic peptide (BNP): forward 5’-GTCAGTCGTTTGGGCTGTAAC-3’, reverse 5’-AGACCCAGGCAGAGTCAGAA-3’; glyceraldehyde 3-phosphate dehydrogenase (GAPDH): forward 5’-TGCAGTGGCAAAGTGGAGATT-3’, reverse 5’-TCGCTCCTGGAAGATGGTGAT-3’. The initial denaturation phase was 5 min at 95°C followed by an amplification phase detailed as following: denaturation at 95°C for 10 s; annealing at 60°C for 30 s; elongation at 72°C for 30 s; detection at 72°C for 40 cycles. Relative expression was calculated with the 2^-ΔΔCt^ method.

### Protein extraction and immunoblot analysis

Total protein extracts were prepared from several pieces of LV frozen samples obtained from the whole hearts of 32-week-old mice (NAGLU^-/-^, n = 5 and WT, n = 5). LV pieces were disrupted by tissue homogenization (Tissue Master 125, OMNI Int.) and lysed in a buffer containing 150 mM NaCl, 50 mM Tris-HCl, pH 7,5, 1 mM EDTA, 1% v/v NP-40, 0.5% w/v deoxycholate, 10 mM NaF, 10 mM Na2pyrophosphate, 2 mM phenylmethylsulfonyl fluoride (PMSF), 2 oridehleupeptin, 2 eptin,aprotinin. Lysates were incubated on ice for 15 min, and then centrifuged at 13000 rpm for 30 min at 4°C. The protein concentration of lysates was measured using a dye-binding protein assay kit (Bio-Rad) and a spectrophotometer reader at a wavelength of 595 nm. Immunoblotting was performed using commercially available antibodies: α-actinin (1:1000, mouse monoclonal, Sigma-Aldrich), α-smooth muscle actin (α-SMA) (1:1000, mouse monoclonal, Sigma-Aldrich), Beclin1 (BCN1) (1:500, rabbit polyclonal, Santa Cruz), calcium/calmodulin-dependent protein kinase II (CaMKII) (1:1000, mouse monoclonal, Santa Cruz), connexin43 (Cx43) (1:500, rabbit polyclonal, Santa Cruz), lipidated isoform of LC3 (LC3 II) (1:500, rabbit polyclonal, Novus Biological), lysosome-associated membrane protein 2 (LAMP2) (1:500, rabbit polyclonal, Pierce), tubulin (1:1000, mouse monoclonal, Sigma-Aldrich). Secondary antibodies were purchased from Amersham Life Sciences Inc and used at concentration of 1:3000. Bands were visualized by enhanced chemiluminescence (ECL; Amersham Life Sciences Inc.) according to the manufacturer’s instructions, and were quantified using densitometry. Each experiment was separately repeated at least three times, and densitometric quantification was normalized versus tubulin protein levels.

### Statistical analysis

Data are expressed as means ± standard deviation (SD). Statistical significance between groups was assessed by Student's t test. For all analyses, a minimum value of p<0.05 was considered significant. The survival curves were constructed using Kaplan-Meier probability estimation, and survival was compared using a log rank survivor function. All the analyses were performed with GraphPad Prism version 5.01 (GraphPad Software).

## Results and Discussion

### NAGLU^-/-^ mice show reduced cardiac function and valvular defects

**Fig 1:**
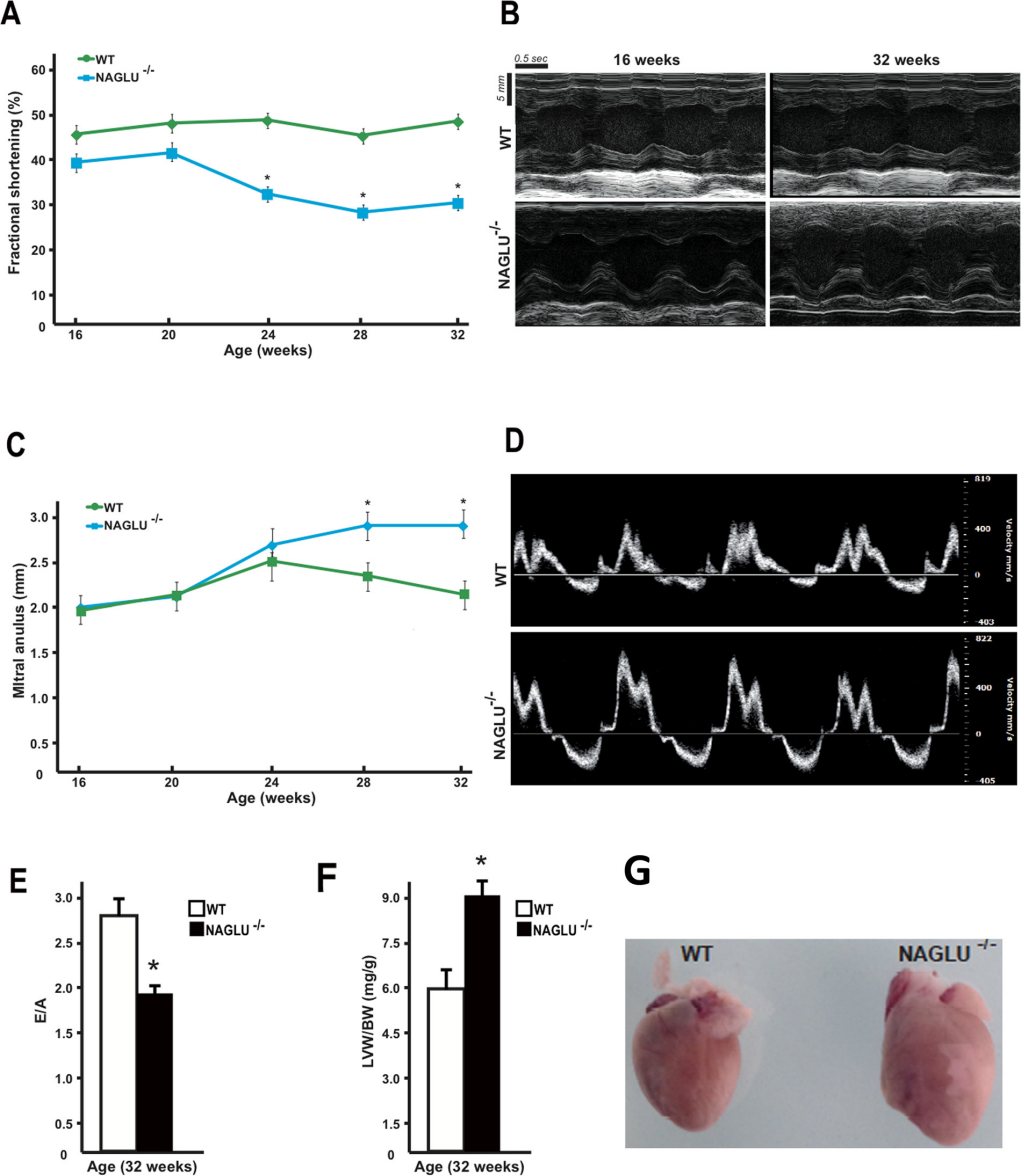
Echocardiographic and morphometric analysis of NAGLU^-/-^ mice. **A**. Cumulative data of % fractional shortening (FS%) of WT and NAGLU^-/-^ mice from 16 weeks to 32 weeks of age (*p<0.05). **B**. Representative M-mode echocardiographic tracings of WT and NAGLU^-/-^ mice at 16 weeks and 32 weeks of age. **C**. Cumulative data of mitral annulus dimension (mm) of WT and NAGLU^-/-^ mice from 16 weeks to 32 weeks of age (*p<0.05). **D**. Doppler analysis of mitral valve regurgitation of WT and NAGLU^-/-^ mice at 32 weeks of age. **E**. Bar graphs showing mitral inflow E-wave to A-wave ratio in WT and NAGLU^-/-^ mice at 32 weeks of age (*p<0.05). **F**. Bar graphs showing left ventricle weight to body weight ratio (LVW/BW) in WT and NAGLU^-/-^ mice at 32 weeks of age (*p<0.05). **G**. Representative gross morphology of whole hearts from 32-week-old WT and NAGLU^-/-^ mice.

The progressive accumulation of undegraded GAGs in MPS results in multiple organ system dysfunctions that vary depending on the particular GAG deposited and the specific enzyme deficiency. Cardiac involvement has been reported in MPS diseases, although it has been better characterized for MPS I, II, and VI, and it significantly contributes to early mortality of these patients [2]. The cardiac causes of death include heart failure, sudden death from arrhythmias, pulmonary hypertension, and coronary occlusion [21–24]. In order to investigate cardiac involvement in MPS IIIB, we evaluated heart morphology and cardiac function in the murine model of MPS IIIB (NAGLU^-/-^) compared to wild-type (WT) mice starting from 16 to 32 weeks of age by serial echocardiograms over time. Interestingly, a decreased percentage of LV fractional shortening (FS%) was observed in NAGLU^-/-^ mice compared to WT animals from 16 weeks to 32 weeks of age (Fig 1A and 1B). A significant reduction in LV FS% started from 24 weeks of age and was maintained up to 32 weeks of age.

Since cardiac valve pathology is the most prominent cardiac manifestation (60–90%) of patients with MPS [2, 6–9, 24], we sought to determine heart valve morphology and dimensions by echocardiography in both NAGLU^-/-^ and WT mice as a function of age at serial time points. Although no differences were found in the right chamber valves and aortic root dimensions between NAGLU^-/-^ and WT hearts at different time points, NAGLU^-/-^ mice exhibited a significant increase in the aortic annulus dimension and mitral annulus dimension and a progressive elongation and thickening of the anterior mitral valve leaflet compared to WT mice (Fig 1C). Accordingly, Doppler analysis of the mitral flow showed that NAGLU^-/-^ mice at 32 weeks of age exhibited a significant downward regurgitant jet at the mitral orifice compared to WT mice (Fig 1D). Moreover, severe mitral regurgitation was confirmed by the reduction of the mitral inflow E-wave to A-wave ratio observed in NAGLU^-/-^ mice at 32 weeks of age compared to age-matched WT (Fig 1E). Consistently with our findings, a retrospective study on 99 patients with MPS reports that mitral regurgitation occurs more frequently in types I, II and III while aortic regurgitation is more common in MPS II and IV, and both mitral and aortic regurgitation showed a positive association with age [7].

Finally, a significant increase in the LV weight (LVW) to body weight (BW) ratio (LVW/BW) was detected in 32-week-old NAGLU^-/-^ mice compared to WT mice (Fig 1F) along with a marked increase of heart dimension (Fig 1G). Reports of ventricular dilatation in MPS are still limited [24–25]. Dilatation in MPS VI patient group was explained mainly by mitral-valve regurgitation [9]. Primary regurgitation causes LV remodeling and dilatation. Generally MPS patients exhibit an increase in geometric features that has been ascribed in part to the compensatory mechanism caused by the mitral regurgitation and in part because of the accumulation of storage material [9].

To assess whether the observed differences in cardiac structures between NAGLU^-/-^ and WT mice would translate in survival benefit, survival rate was monitored for over 30 weeks after birth in both groups of mice. Compared to WT mice, NAGLU^-/-^ mice exhibited a significantly lower survival with increased mortality observed in particular after 25 weeks of age (S1 Fig). Overall, these results indicate that NAGLU^-/-^ mice develop cardiac dysfunction in age-dependent manner influencing the survival rate of murine MPS IIIB.

### NAGLU^-/-^ mice show cardiac valve thickening

**Fig 2:**
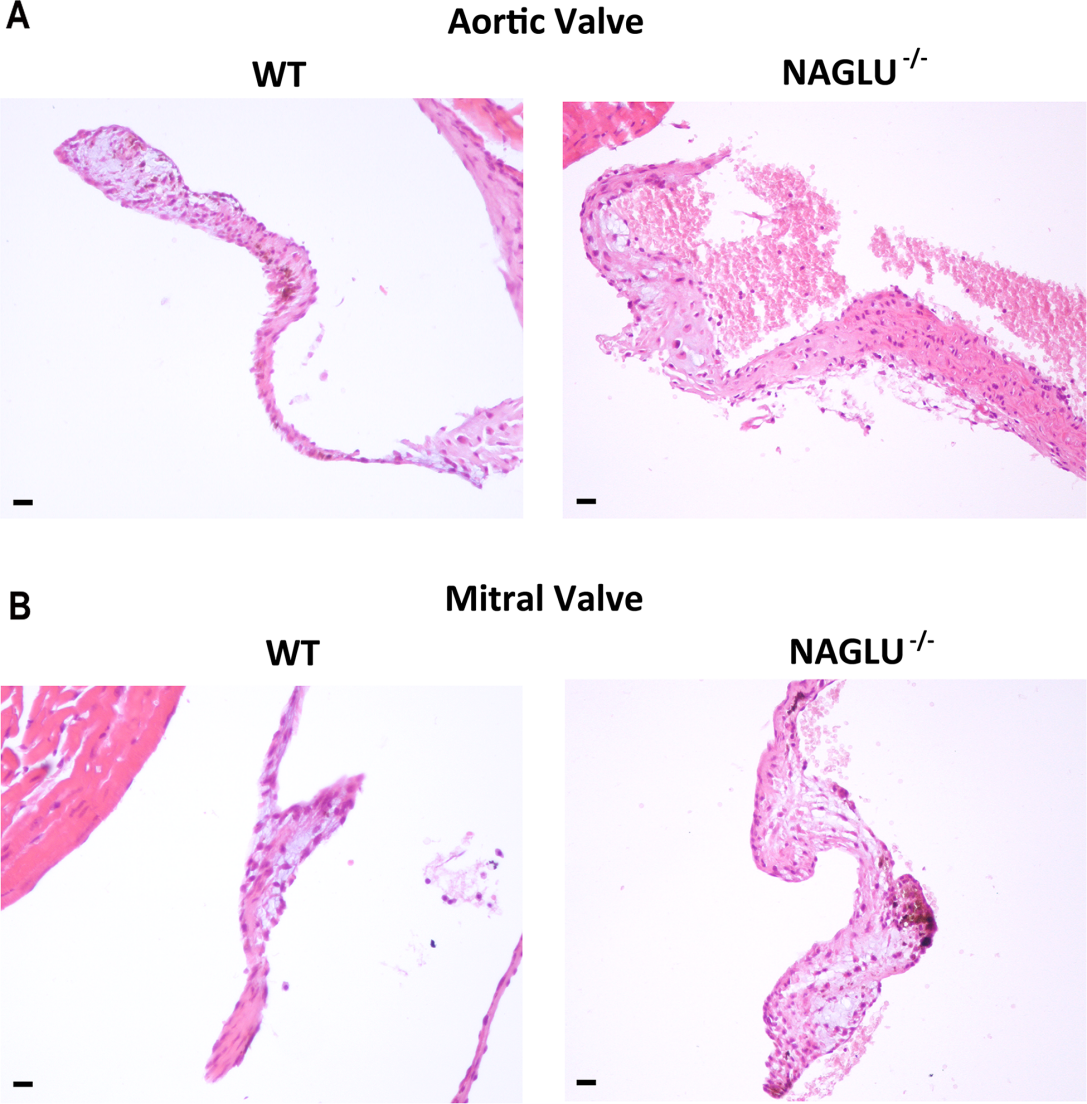
Histological analysis of cardiac valves thickening. **A**. Representative images of haematoxylin and eosin (H&E) of aortic valve sections from WT and NAGLU^-/-^ hearts at 32 weeks of age (20x magnification). **B**. Representative images of haematoxylin and eosin (H&E) of mitral valve sections from WT and NAGLU^-/-^ hearts at 32 weeks of age (20x magnification). Scale bars: 20 μm.

The echocardiographic findings prompted us to carry out a histological analysis of cardiac valve structure. The analysis of both aortic and mitral valve morphology in NAGLU^-/-^ and WT mice showed aortic and mitral valve defects in NAGLU^-/-^ at 32 weeks of age (Fig 2). In particular, NAGLU^-/-^ mice exhibited a significant aortic (Fig 2A) and mitral (Fig 2B) cuspid thickening as shown by H&E staining. Mitral and aortic valve thickening has been reported in canine MPS VII [26] as well as in a MPS I and VI mouse models [27–28]. Our results, in agreement with echocardiographic and Doppler analyses, indicate that the alterations in the valve morphology in NAGLU^-/-^ mice are associated with a progressive reduction in cardiac function over time and that abnormal valve morphology and function produce both diastolic and systolic dysfunctions, suggesting that severe mitral regurgitation is the "primum movens" of cardiac dysfunction in MPS IIIB mice.

### NAGLU^-/-^ mice show myocardial vacuolization, inflammation, and fibrosis

**Fig 3:**
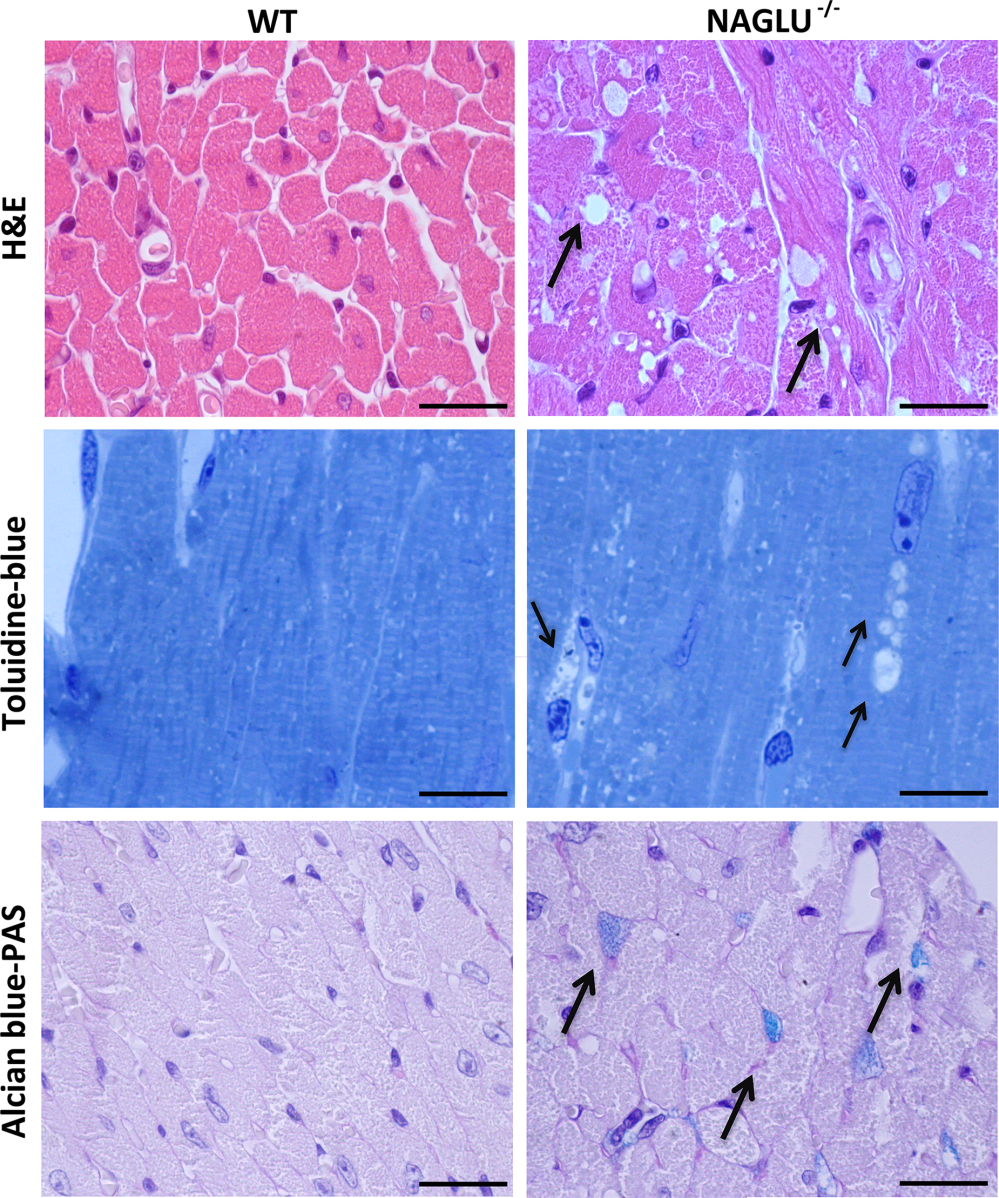
Histological analysis of myocardial vacuolization. Representative H&E, Toluidine blue and Alcian blue-PAS staining of LV tissues form heart of 32-week-old WT and NAGLU^-/-^ mice (100x magnification) shows vacuolization and deposition of HS into vacuoles. Arrows indicate vacuoles within myocardial fibers in the H&E (upper panel) and toluidine blue (middle panel), and HS accumulation in the Alcian blue-PAS staining (lower panel). Scale bars: 20 μm.

In order to further investigate the features of cardiac disease in the MPS IIIB mouse model, a histopathological analysis of the myocardium of NAGLU^-/-^ mice was performed. This analysis revealed a significant increase in myocardial fiber vacuolization compared to WT mice, as shown by H&E staining (Fig 3). Lysosomal storage and cellular vacuolization was also qualitatively evaluated by bright-field microscopy on plastic embedded semithin section stained by toluidine blue revealing at higher resolution the presence of vacuoles within the myocardial fibers of MPS IIIB mice (Fig 3). Furthermore, Alcian blue-PAS staining shows the accumulation of HS in the myocardial vacuoles of NAGLU^-/-^ mice as indicated by areas of blue color (Fig 3). The only GAG accumulated in NAGLU^-/-^ mouse tissues is represented by HS due to the specific enzymatic defect that characterizes MPS IIIB disease from other MPS syndromes in which accumulation of GAGs is mixed [1, 11]. Thus, the storage of HS may represent the main cause of valve defects and myocardial dysfunction in MPS IIIB mice.

**Fig 4:**
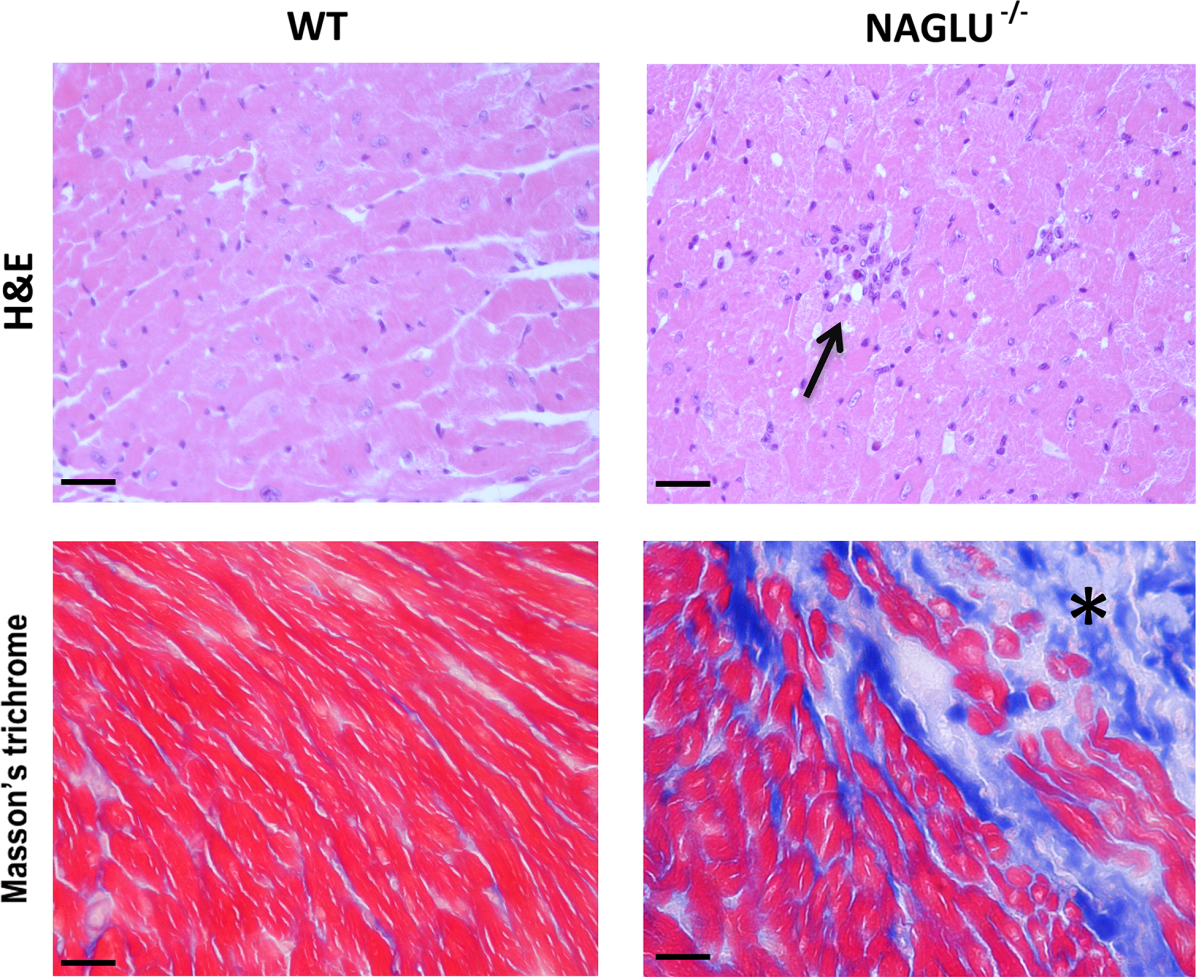
Histological analysis of myocardial inflammation and fibrosis in NAGLU^-/-^ mice. Representative H&E staining for inflammatory infiltrate and Masson's trichrome staining for fibrosis in LV tissues from WT and NAGLU^-/-^ mice (40x magnification). Arrow indicates inflammatory infiltrates within myocardial fibers and asterisk (*) indicates fibrosis within myocardial fibers. Scale bars: 20 μm.

To investigate whether affected myocardium of NAGLU^-/-^ mice presents increased inflammation and fibrosis, features often related to the cardiac failure, the presence of inflammatory infiltrates and fibrosis was evaluated respectively by H&E and Masson's trichrome staining (Fig 4). NAGLU^-/-^ mice exhibited a significant recruitment of inflammatory cells within the myocardium labeled by the specific inflammatory markers CD3, CD4, CD8 and CD68 (S2 Fig). Moreover, the staining with CD68 confirmed the presence of vacuoles within the macrophages (arrows in S2 Fig), in accordance with literature data [11]. Concerning fibrosis of affected mice, collagen deposition was localized near the valves and at lower extent in the myocardium (S3 Fig). Many reports deal with an abnormal content or structure of collagen in cardiac tissues of MPS animal models and patients [2, 8, 25–26, 28–32]. Our results are consistent with previous findings showing myocardial fibrosis in the murine models of MPS I [28] and MPS II [29], and the deposition of large amounts of collagen in the valves and myocardium of patients affected by Hurler syndrome [30].

Overall, these results demonstrate, for the first time, that NAGLU gene deletion causes in the murine model of MPS IIIB specific myocardial structure alterations due to HS vacuolization, increased inflammation and fibrosis.

### NAGLU^-/-^ mice show increased levels of cardiac failure markers

**Fig 5:**
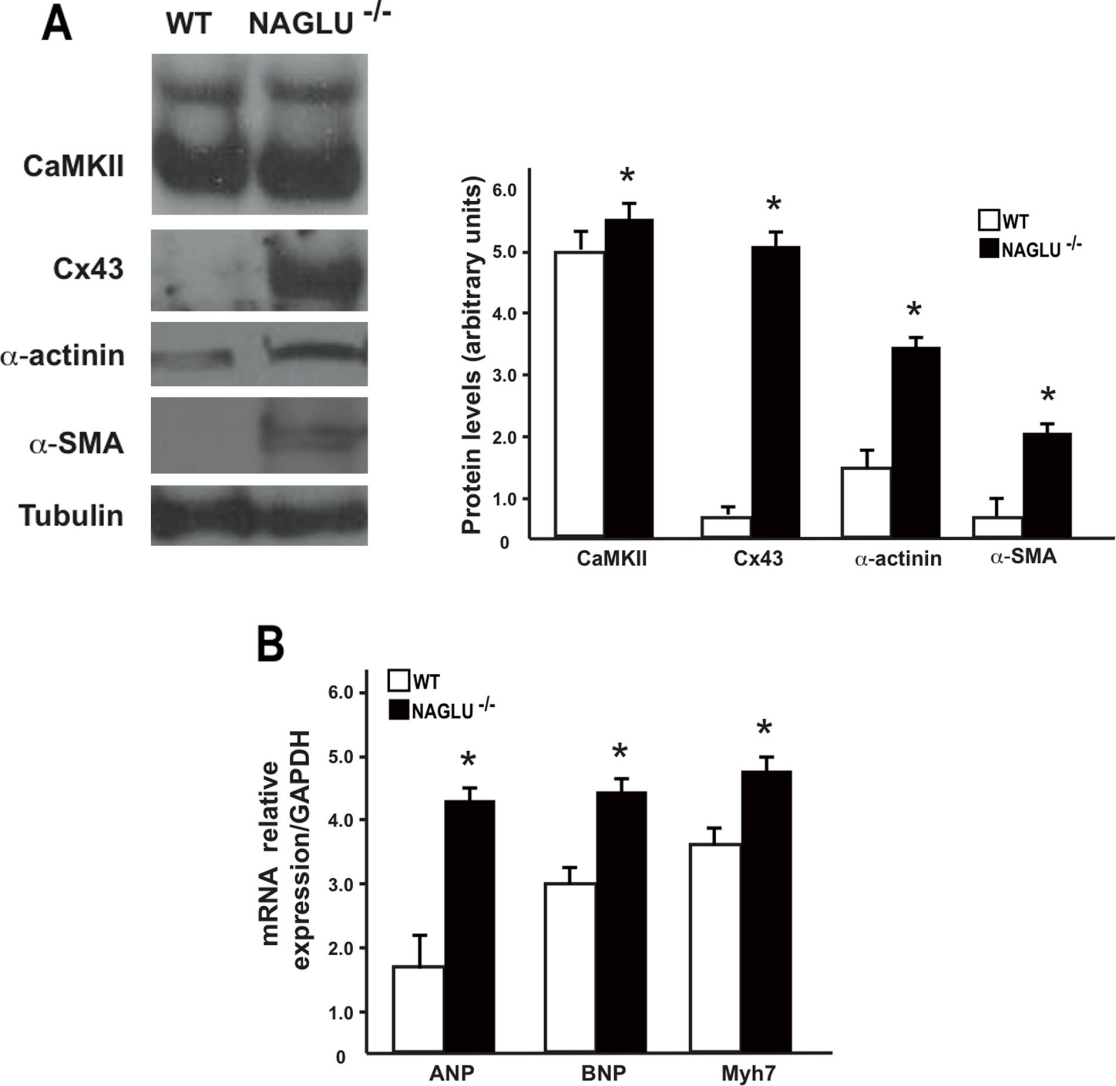
Biochemical evaluation of cardiac failure markers in NAGLU^-/-^ mice. **A**. Representative immunoblot and densitometric analysis of CaMKII, Cx43, α-actinin and α-SMA protein levels in WT and NAGLU^-/-^ heart samples at 32 weeks of age (*p<0.05). **B**. Real-time PCR analysis of ANP, BNP and Myh7 mRNA levels in WT and NAGLU^-/-^ hearts at 32 weeks of age (*p<0.05).

In order to confirm the ecocardiographic and histopathological data of the cardiac involvement in the MPS IIIB mouse model at the molecular level, biochemical analyses of LV samples from NAGLU^-/-^ and WT mice were carried out. First, we found an enhanced expression of hypertrophy and fibrosis markers in heart tissues from NAGLU^-/-^ mice compared to WT littermates, as demonstrated by increased myocardial protein levels of CaMKII [33], Cx43 [34], α-actinin [35] and α-SMA [18] (Fig 5A). Moreover, LV samples from NAGLU^-/-^ mice were characterized by a significant increase in the mRNA levels of ANP, BNP and Myh7 (Fig 5B), hallmarks of cardiac dysfunctions and failure [36–37].

Overall, these results are consistent with the cardiac dysfunctions detected by echocardiography and histology in affected mice, and demonstrate that NAGLU gene deletion causes specific myocardial structure alterations over time leading to cardiac failure.

### Autophagy impairment in the heart of NAGLU^-/-^ mice

**Fig 6:**
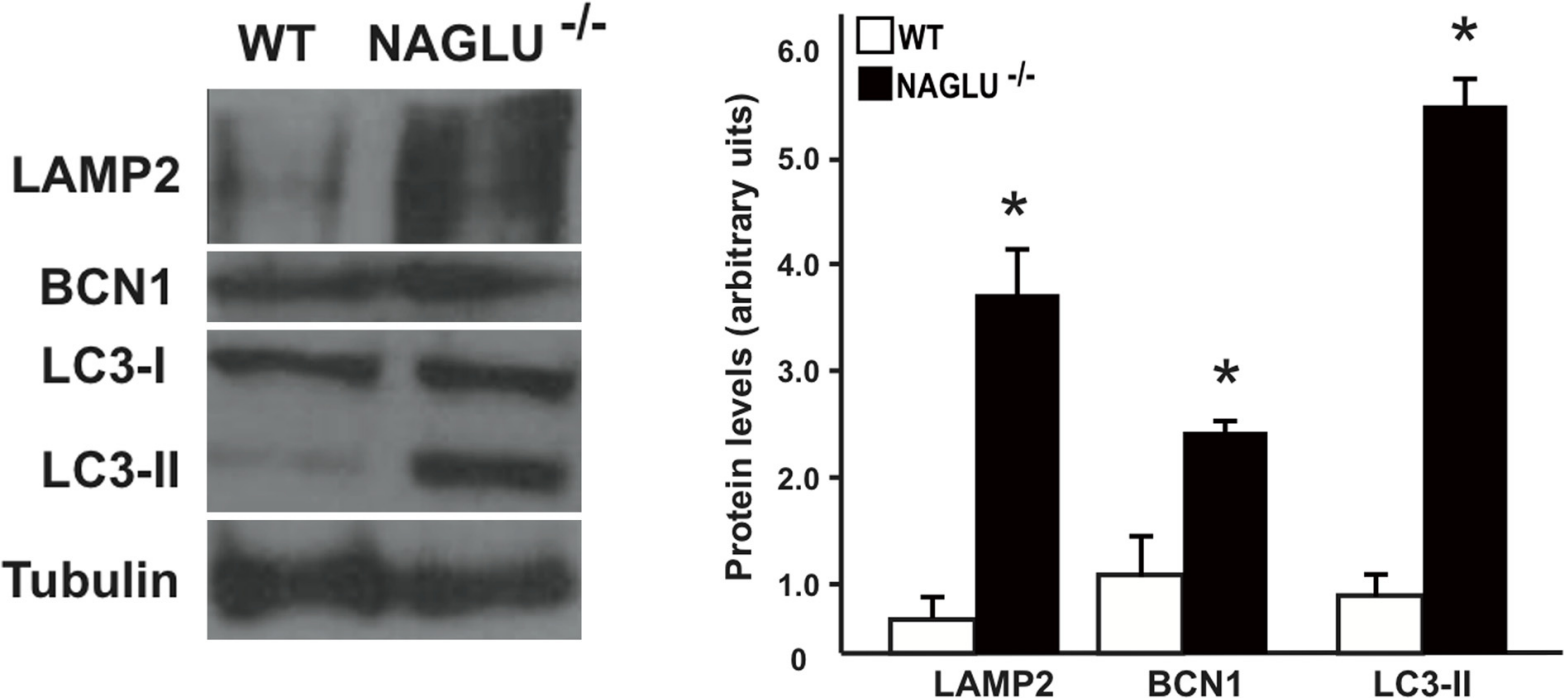
Evaluation of lysosomal and autophagy markers in NAGLU^-/-^ hearts. Representative immunoblot and densitometric analysis of LAMP2, BCN1 and LC3-II protein levels in WT and NAGLU^-/-^ hearts at 32 weeks of age (*p<0.05).

Alterations in the autophagic process have recently been suggested as a pathogenic mechanism common to a variety of lysosomal storage diseases including some MPS IIIA and VI types [12–13]. However, the block of autophagy in MPS has never been studied in connection with cardiac problems. Here, we investigated whether an impairment of autophagy might be associated to cardiac dysfunctions in NAGLU^-/-^ mice together with the lysosomal defects. To this purpose, we first investigated cardiac lysosomal involvement in NAGLU^-/-^ mice by evaluating the protein levels of the lysosomal marker LAMP2 [38]. Cardiac levels of LAMP2 were significantly increased in NAGLU^-/-^ mice compared to WT mice, as shown by Western blotting analysis (Fig 6). We found in LV samples of NAGLU^-/-^ mice an increased expression of Beclin1 (BCN1, homologue of yeast ATG6), a protein of the Class III phosphatidylinositol 3 kinase (PI3K) complex that mediates the early phase of autophagy [39] (Fig 6). BCN1 protein is involved in the formation of autophagosome. The microtubule-associated protein I light chain (LC3-I, homologue of yeast ATG8) is also implicated in this process; LC3-I is cleaved at its carboxy-terminal and further modified to the lipid-conjugated LC3-II which is associated to autophagosome membranes [40]. LC3-II levels are reflective of autophagosome abundance. Western blotting analysis of mouse heart samples showed that LC3-II expression levels were significantly increased in cardiac samples from NAGLU^-/-^ mice compared to WT littermates (Fig 6). These results indicate that the autophagic process is dysfunctional in the heart of NAGLU^-/-^ mice, thus suggesting that dysregulated autophagic capacity can have a detrimental impact in heart tissue, and might represent an important factor contributing to the cardiac disease in MPS IIIB. Indeed, in the heart of NAGLU^-/-^ mice, lysosomal accumulation of HS, due to NAGLU enzyme deficiency, activates autophagy, as demonstrated by increased BCN1 expression levels, but the formed autophagosome cannot be cleared, as shown by the enhanced expression of LC3-II in cardiac samples of NAGLU^-/-^ mice as compared to WT mice. However, further investigations are needed to establish whether the substrate accumulation is the primary mediator of abnormal lysosomal autophagy, and whether the accumulation of autophagosomes rather than the block of the autophagosome-lysosome fusion is responsible for autophagy impairment in the heart of affected mice.

## Conclusions

This study addresses for the first time the cardiac dysfunction in MPS IIIB. Our results demonstrate that NAGLU^-/-^ mice develop abnormal valve morphology and function in an age-dependent manner in association with increased myocardial vacuolization, inflammation and fibrosis. Furthermore, our data showing a dysregulated lysosomal autophagy in the cardiac tissues of NAGLU^-/-^ mice suggest that an abnormal autophagic pathway may represent an important factor contributing to cardiac disease in the murine model of MPS IIIB, and presumably this holds true also for human patients. An in-depth understanding of the pathophysiology of heart disease in MPS IIIB is essential for adequate evaluations and timely management of these patients to improve clinical outcomes and quality of life. Our findings could be relevant in the screening of new therapies for the treatment of MPS IIIB disease.

## Supporting Information

S1 Fig Kaplan-Meier cumulative survival analysis of WT and NAGLU^-/-^ mice (*p<0.05). (TIF)

S2 Fig Immunostaining of individual immune cells in serial sections of NAGLU^-/-^ heart. Sections were stained with anti-sera raised against CD3, CD4, CD8 and CD68. All images were acquired at 40x magnification. Scale bars: 20 μm.
(TIF)

S3 Fig Localization of the fibrotic area near the valves of NAGLU^-/-^ heart. Lower magnifications (4x and 10x) of NAGLU^-/-^ Masson's trichrome staining shown in Fig 4. Scale bars: 200 μm.
(TIF)
