# Supplementary figures and images for: The Murine Model of Mucopolysaccharidosis IIIB Develops Cardiopathies over Time Leading to Heart Failure
Source: PLoS One. 2015 Jul 6;10(7):e0131662. doi: 10.1371/journal.pone.0131662 (PMC4493027; doi:10.1371/journal.pone.0131662)

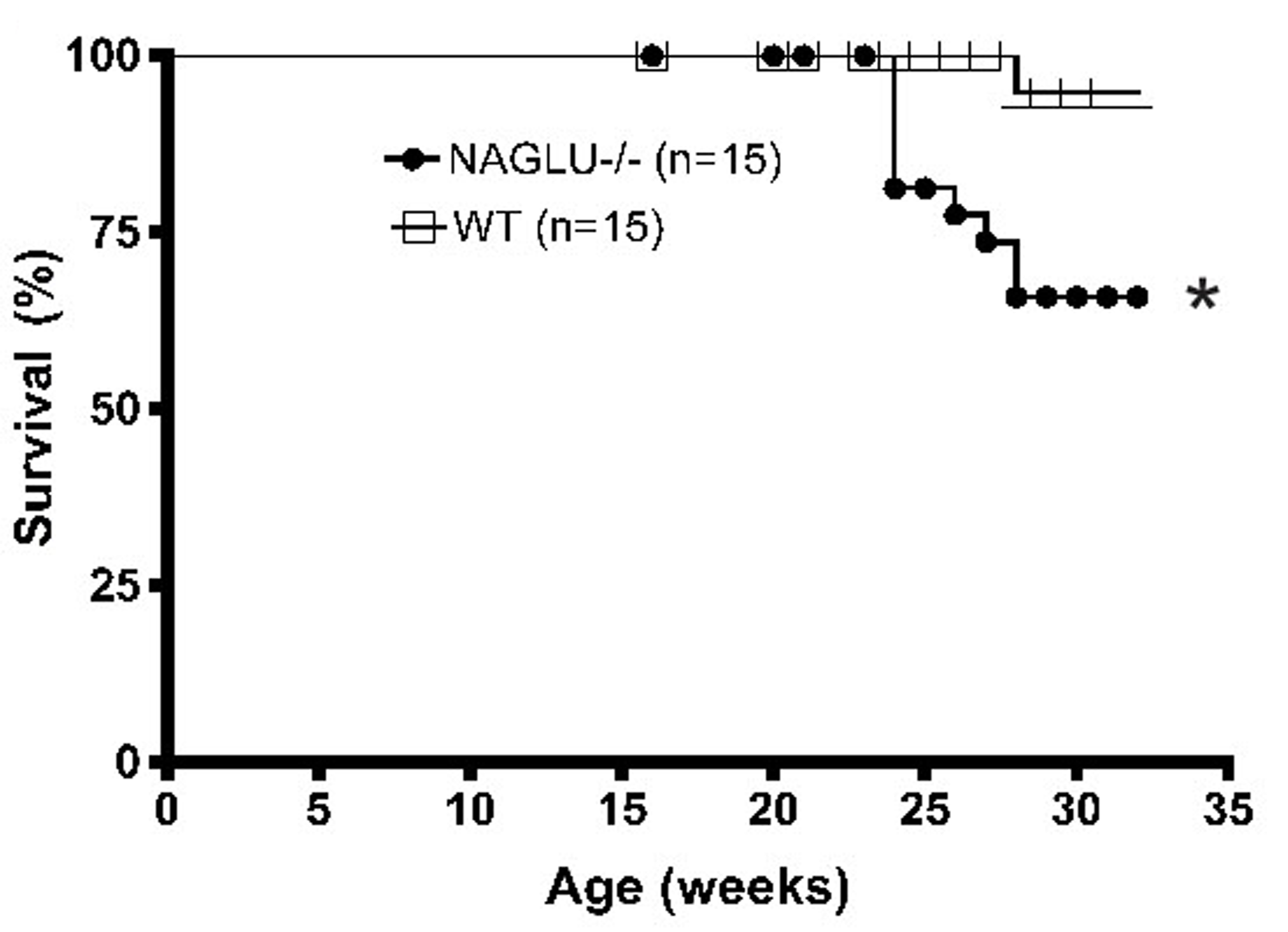

Supplement: S1 Fig — (TIF) [file pone.0131662.s001.tif]

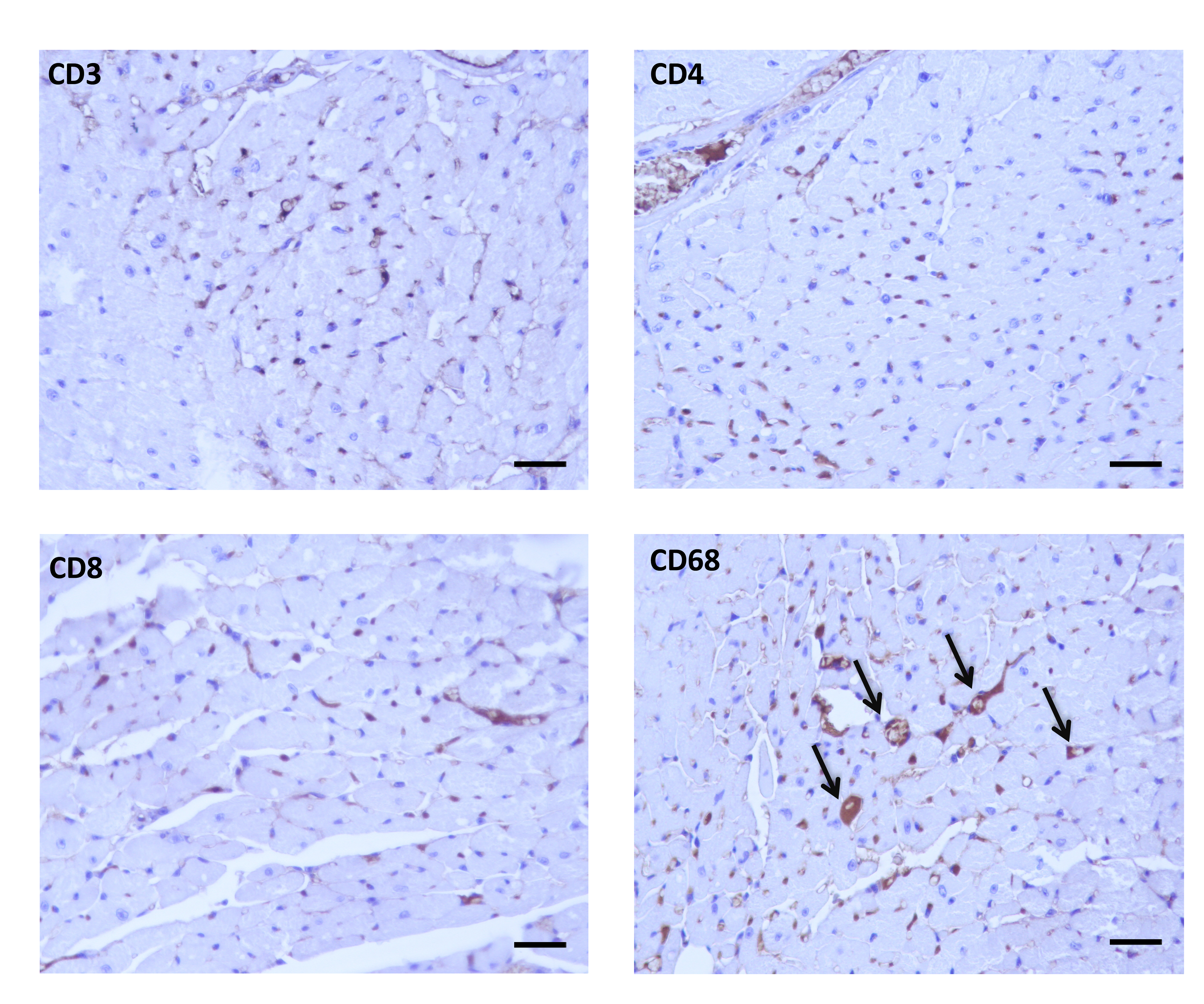

Supplement: S2 Fig — Sections were stained with anti-sera raised against CD3, CD4, CD8 and CD68. All images were acquired at 40x magnification. Scale bars: 20 μm. (TIF) [file pone.0131662.s002.tif]

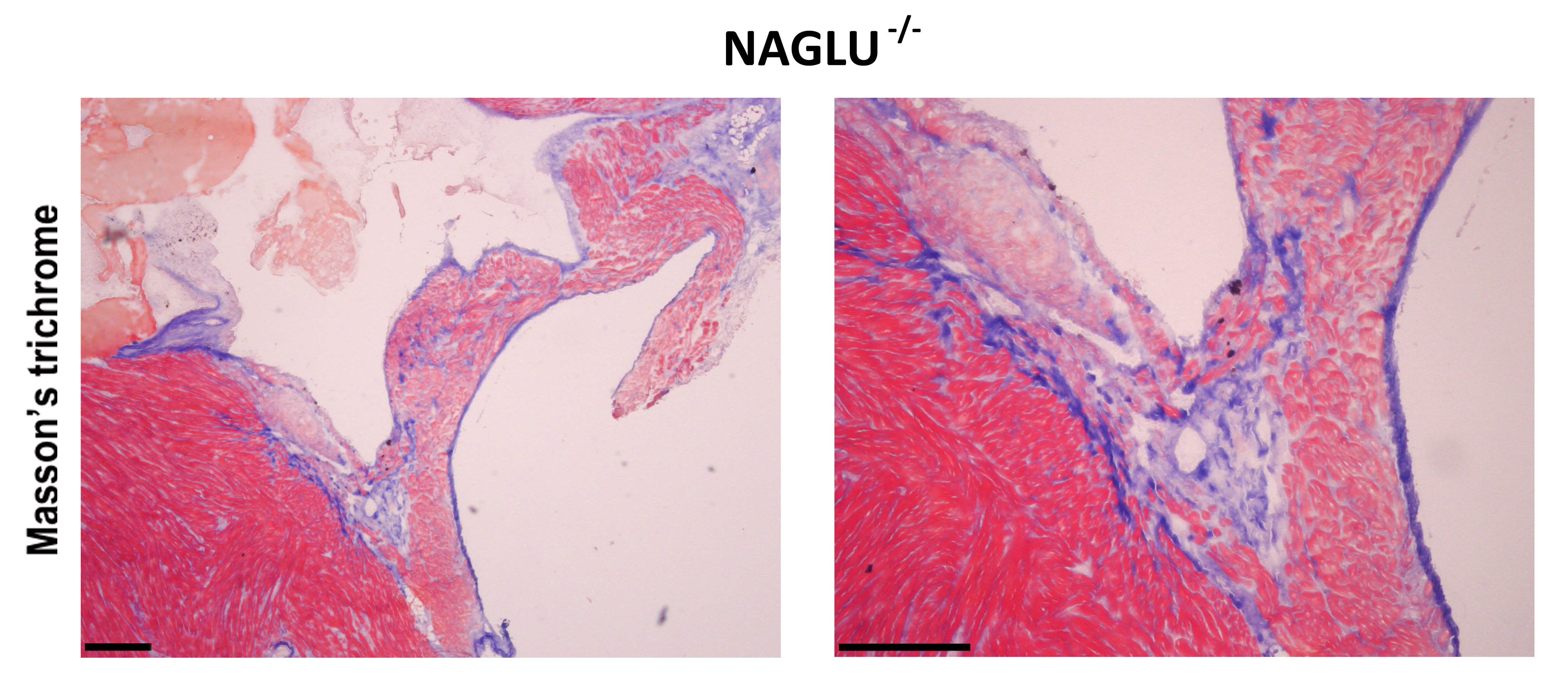

Supplement: S3 Fig — Lower magnifications (4x and 10x) of NAGLU-/- Masson's trichrome staining shown in Fig 4. Scale bars: 200 μm. (TIF) [file pone.0131662.s003.tif]
